# DoE of *fed-batch* processes – model-based design and experimental evaluation

**DOI:** 10.1186/1753-6561-5-S8-P46

**Published:** 2011-11-22

**Authors:** Onur Sercinoglu, Oscar Platas Barradas, Volker Sandig, An-Ping Zeng, Ralf Pörtner

**Affiliations:** 1 Institute of Bioprocess and Biosystems Engineering, Hamburg University of Technology, Hamburg, D-21073, Germany; 2 ProBioGen AG, Berlin, D-13086, Germany

## Background

Experimental process-development and optimization is expensive and time-consuming. Real optimization by means of design of experiments involves data generation before optimization can be aimed for. This can make the way from process development to process establishment even harder, since academia or start-up research facilities might not have the possibility to generate these data. Furthermore, bioprocesses involving mammalian cells deal with many critical variables; processes are not only carried out batch wise, but increasingly in *fed-batch* mode with desired feeding profiles.

The use of DoE tools in combination with an appropriate growth model might allow the experimenter to develop and to test *fed-batch* strategies *in silico*, before experiments are carried out in the laboratory.

In our work, an unstructured model for mammalian cell culture was used for simulation. Kinetic parameters were derived from a small number of shake-flask experiments. The model was tested for data generation on common *fed-batch* strategies. By means of design of experiments strategies, relevant conditions were selected and experimentally tested. In this way, suitable *fed-batch* strategies for mammalian cell lines are evaluated *in silico* before bioreactor experiments are to be performed. This results in a significant reduction in the number of experiments during process development for mammalian cell culture.

## Concept – *fed-batch* Analyzer -

A tool was developed in Mathworks‘ Matlab for simulation of batch and *fed-batch* cultivations of mammalian cells. This program was created as a GUI (Guided User Interface), in which the user will be guided through the process of developing a *fed-batch* strategy. Growth models can be selected as well as different *fed-batch* modes. For a manageable comparison of *fed-batch* strategies, *in silico* experiments can be planned by design of experiments (DoE) and initial conditions as well as cell-line-specific kinetic parameters can be obtained from a reduced number of small-scale experiments e.g. shake flasks. The following steps will explain the procedure for *in silico* experimentation during the development of a *fed-batch* strategy for the human production cell line AGE1.hn (ProBioGen AG). Afterwards, one process condition will be chosen and tested in the laboratory.

### Step 1: Define the process model

Growth models [1,2] have been predefined for operation of the tool. However, the user can modify or include new models according to her/his expertise and knowledge of the cell line. For determination of model parameters, a reduced number of experiments in small scale (e.g. shake flasks), is to be performed.

### Step 2: Load the model

### Step 3: Choose a feeding strategy

Simple feeding strategies have been predefined in order to make the transfer of *in silico* results into laboratory experimentation easier. Constant feed, linear feed, exponential feed and step feed can be selected. New feeding profiles can be added to the program to improve its possibilities.

### Step 4: Create design

The design (e.g. full factorial design) can be either planed within the program (MATLAB Statistics Toolbox) or loaded from other DoE tools (e.g. Design Expert). In order to define the central point for the design, data from batch experiments can be used.

### Step 5: Run design

The tool conduct the *in silico* experiments and delivers a response. This is normally a variable the user is interested in (IVCD, VCD or product concentration).

### Step 6: Obtain *in silico* results

**Figure 1:**
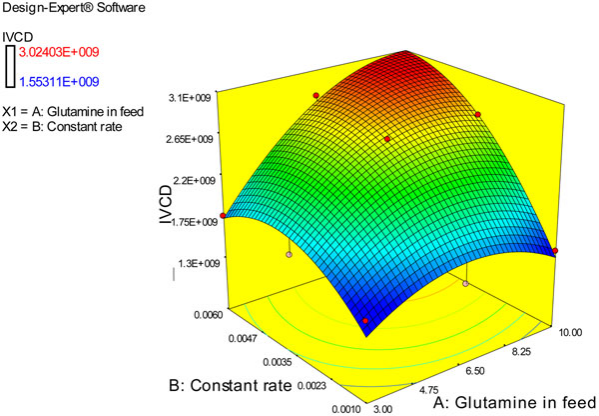
Surface response plan for a feeding strategy with constant feed rate.

For each set of conditions, the tool generates a sheet with virtual curves. For an overview of results and better comparison of the process conditions, a response curve can be generated (see Figure 1).

### Step 7: Implementation in the laboratory

**Figure 2:**
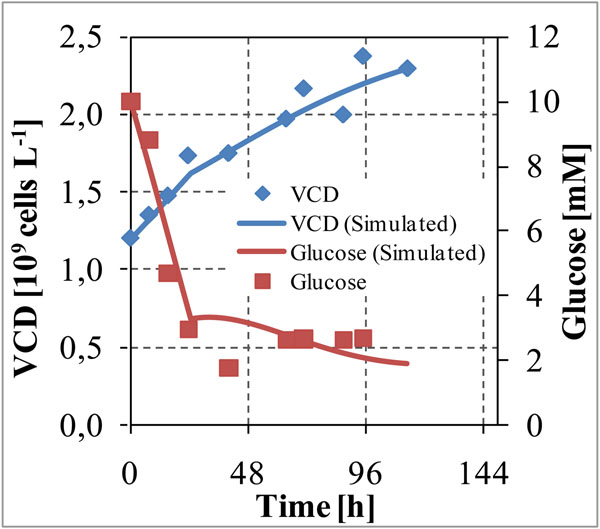
Experimental results after culture of AGE1.HN cells in bioreactor with a constant feed rate.

Following experiment was planned and performed in a 1 L Bioreactor. (Table 1, Figure 2)

**Table 1:** Experimental conditions for validation of a contant feeding strategy.

Parameter	Value
Initial conditions (*batch*)	

Glucose	10 mM
Glutamine	2 mM

*fed-batch* strategy	

Mode	constant
Glucose in Feed	60 mM
Glutamin in Feed	8 mM
Feed Rate	0.059 mL min^-1^
Feed Start	24 h
